# Cardiovascular monitoring systems for aging in place: current perspectives and a novel toilet seat-based system

**DOI:** 10.1093/geroni/igab046.3566

**Published:** 2021-12-17

**Authors:** Carla VandeWeerd, Mitchell Roberts, Lindsey Collins, Erica Sappington, Lydia Poon, Jeff Lowenkron

**Affiliations:** 1 UF Health Precision Health Research Center-The Villages, The Villages, Florida, United States; 2 University of Florida, Tampa, Florida, United States; 3 University of Florida, University of Florida, Florida, United States; 4 The University of Florida, The Villages, Florida, United States; 5 The Villages Health, The Villages, Florida, United States

## Abstract

Cardiovascular disease (CVD) is a leading cause of death. Questions remain as to how older adults, providers and researchers can harness remote patient monitoring (RPM) to maintain/improve cardiovascular health--especially in light of COVID-19 and increased reliance on telehealth. The objective of this study was to understand the perceptions of older adults with cardiovascular challenges and providers surrounding a novel RPM device. The Heart Seat (THS) developed by Casana, is a toilet-seat-based cardiac monitoring device. Focus groups, stratified by gender, were conducted in 2021 by the UF Health Precision Health Research Center (UFIRB202100290) with older (55+) adults (n=36) in The Villages, Florida. Adoption, benefits/concerns, usability, utility and gender differences were explored. One-on-one provider interviews (n=6) explored future utility of THS. The primary benefit of THS noted by providers and older adults was ease-of-use and passive data collection, promoting adherence. Providers considered THS ‘easy-to-use’ and a positive alternative to current RPM devices. While genders' sentiments towards cardiac monitoring devices were similar, males reported having more experience with RPM. Despite this, females reported using cardiac monitoring devices more consistently than males. Therefore, passive RPM may be beneficial for increasing adherence in males. Participants' largest concern surrounding RPM was information sharing, including data monitoring, and security. Providers were also concerned about information sharing, specifically who would receive/monitor and interpret data from RPM. RPM devices should focus on enhancing ease-of-use, catering to user and provider information sharing and data monitoring/interpretation preferences and privacy.

